# Exploring the Role of Volunteer Organizations in Developing Italy’s Community-Based Care Model

**DOI:** 10.5334/ijic.7881

**Published:** 2025-07-02

**Authors:** Federico De Luca, Giuliana Costa, Cristina Masella

**Affiliations:** 1 Department of Management Engineering, Department of Design, Politecnico di Milano, Milan, Italy; 2 Department of Architecture and Urban Studies, Politecnico di Milano, Milan, Italy; 3 Department of Management Engineering, Politecnico di Milano, Milan, Italy

**Keywords:** community-based care, volunteer organizations, primary care, community health center

## Abstract

**Introduction:**

Community-based healthcare models are crucial for reforming primary care delivery and integrating prevention, health promotion, and care services within communities. Volunteer organizations are increasingly recognized for their potential contributions; however, their integration within Italy’s emerging Community Health Center (CHC) model still needs to be explored. This study investigated the role of volunteer organizations in developing Italy’s CHC model by focusing on how these groups can enhance care coordination and community health outcomes.

**Methods:**

A qualitative descriptive approach was employed, combining semi-structured interviews and focus groups with key stakeholders, including CHC managers, primary care directors, social service providers, and volunteer organization representatives in the Piacenza area of Emilia Romagna. Data collection spanned July 2021 to March 2022, and thematic analysis was used to identify core themes related to the integration of volunteer organizations in CHCs.

**Results:**

The study identified four key areas where volunteer organizations contribute: (i) prevention and health promotion, (ii) identifying unmet needs and caregiver support, (iii) collaborative initiatives between CHCs and volunteers, and (iv) creating spaces for teamwork. Despite these contributions, challenges related to organizational coordination, limited operational specialization, and geographic disparities have been noted. Volunteer organizations were found to play a critical role in addressing gaps in community services, yet their involvement in CHC planning and execution varied across territories.

**Discussion:**

Although volunteer organizations have the potential to significantly enhance community-based care, their integration into Italy’s CHC model is hindered by limited coordination, funding constraints, and uneven involvement across regions. Strengthening partnerships, improving operational support, and creating dedicated collaboration spaces are essential to fully leveraging their contributions. Future research should explore strategies for enhancing the sustainability and scalability of volunteer-led initiatives within the CHC framework.

## Introduction

Community-based healthcare represents a promising strategy for reforming primary care delivery. As an integral component of healthcare systems, community-based services include care, prevention, and health promotion, delivered in organizations and facilities with defined healthcare attributes. [1,2,3]. Extensive scholarly research suggests that primary and territorial care services within health systems contribute significantly to improved health outcomes at the population level [4]. Furthermore, these services are recognized by the World Health Organization (WHO) as a fundamental human right [5,6,7]. They are crucial for establishing sustainable partnerships between healthcare professionals, patients, volunteers, and the broader community [8].

Effective primary care systems are defined by four core attributes: first-contact access, long-term person-focused care, comprehensive services, and coordinated care [9,10,11]. The integration of community volunteers within these care settings has the potential to enhance these attributes, fostering a more cohesive and supportive healthcare environment [12]. However, despite their potential contributions, the role of volunteers in primary care remains a relatively underexplored area of academic research [12,13].

Volunteer collaboration in community and hospital settings profoundly impacts patient and caregiver well-being, including positive changes in patients’ health behaviors [14], reduction of hospital admissions, shorter hospital stays, and lower readmission rates [15], as well as improving psychosocial outcomes and increasing self-confidence in managing chronic conditions such as cancer and diabetes [16,17,18]. By assuming routine tasks, volunteers alleviate the workload of paid health and social staff, enabling professionals to focus more effectively on delivering specialized care to those in need [19].

Volunteers in these programs engage in a wide range of activities, such as organizing physical activity sessions [20], providing education on topics such as nutrition, diabetes management, and cardiovascular health, and offering health coaching or peer counseling. Notably, volunteer-led interventions have, in many instances, demonstrated effectiveness comparable to that delivered by trained professionals [21,12].

Beyond the benefits to patients and healthcare systems, volunteering is also advantageous for the volunteers. These benefits include improved self-reported health and well-being, reduced depression levels, increased life satisfaction, fewer functional limitations, and lower mortality rates [22]. Volunteer training programs further contribute to positive outcomes by fostering protective factors. For instance, peer support volunteers in chronic disease areas often report enhanced self-confidence in managing their condition and experience a decrease in feelings of depression following training [23,24,25].

Volunteering also holds a significant economic value for healthcare systems. Studies focusing on hospital and community-based volunteers indicate that their benefits outweigh the costs of managing volunteer programs [26,27]. Voluntary organizations are crucial in enhancing patient satisfaction and supporting healthcare professionals facing staff shortages and financial constraints [28]. Furthermore, they often provide new perspectives on organizational activities and mitigate operational blind spots [29]. However, several studies on volunteer engagement [30,31] in health activities have highlighted the limited involvement of healthcare professionals, stressing that the contributions of volunteers and their potential for driving innovation and anticipating community needs are often undervalued by professionals. However, most volunteers possess local knowledge that is vital for creating a conducive social environment and improving multiple health determinants [32]. Being deeply familiar with the daily lives of community members, volunteers can reinforce social bonds within the regions they serve [33].

Despite the well-documented advantages for patients, volunteers, and the broader healthcare system, the involvement of volunteers in primary care remains limited, and evaluations of these programs are scarce [12,34]. To strengthen the role of voluntary organizations, the Italian National Health Service (INHS) highlighted their significance in the primary care reform enacted through Law No. 296 on December 27, 2006, further reinforced by the Parliament in 2021. This reform introduced a new primary care organizational model, the “Community Health Center” (CHC), also named “Medical Home” in specific contexts [35]. Called “Case della Salute” until 2021, these centers were later rebranded with little organizational changes as “Case della Comunità.” They are territorial structures designed to strengthen the primary care system. A key objective of this model is the integration of voluntary organizations into community-based services. Through collaborative partnerships with local volunteers, CHCs aim to enhance the overall experiences, outcomes, safety, and care efficiency of patients and healthcare professionals, with the ultimate goal of supporting individuals in maintaining longer and healthier lives within their communities [36].

The foundational principles of the CHC model include a focus on team-based care, a patient-centric approach across the lifespan, improved access to care, provision of comprehensive services, coordination of services across the healthcare and welfare sectors, quality and safety assessment based on evidence-based practices, and the availability of care beyond regular hours. These principles aim to deliver high-quality, effective care while also controlling healthcare costs by reducing inappropriate hospital admissions and minimizing the unnecessary use of hospital resources. [37–38]. The CHC model is fundamentally rooted in a community-based approach in primary care settings.

This study examines the critical role of third-sector organizations in implementing the CHC model in Italy. Specifically, it addresses the following research question: *What factors facilitate the seamless integration of voluntary organizations into CHC implementation?* The primary objective is to outline the framework of the research project, providing valuable insights that can serve as a resource for other primary care organizations that aim to incorporate volunteers into their operational structures. It is essential to emphasize the limited amount of research conducted in Italy on the role of volunteering in healthcare. This study aims to contribute to this field by identifying and exploring new avenues for research and development in this context.

## Methodology

This research is part of a broader project exploring how CHCs can be transformed and assume a role beyond being healthcare facilities, promoting community health to address social vulnerabilities. This study utilized a qualitative descriptive approach, as defined by Sandelowski [39], within a pragmatic research framework [40] to investigate the role of volunteer organizations in implementing CHCs in the Piacenza area, Emilia Romagna Region, Italy. During the first wave of the COVID-19 pandemic, Piacenza was one of Italy’s hardest-hit provinces, experiencing more than a 100% increase in mortality between March and May 2020 compared with the previous year [41]. Early reports [42] highlighted significant weaknesses in the local healthcare system, particularly in managing the surge of patients and the strain on territorial and home care services. Healthcare in the Piacenza area is managed by the Local Health Authority (LHA), whereas social services are provided by the municipality. The Emilia Romagna Region is notable for fully developing the CHCs model, in contrast to most others that have not yet implemented it.

This research was conducted as part of an interdepartmental project involving four researchers: two management engineers, a sociologist, and a service designer. Fieldwork was conducted in Piacenza province from July 2021 to March 2022, despite the challenges posed by the COVID-19 pandemic. The research unfolded through several phases, including semi-structured interviews designed for primary care contexts [43].

**Figure 1:**
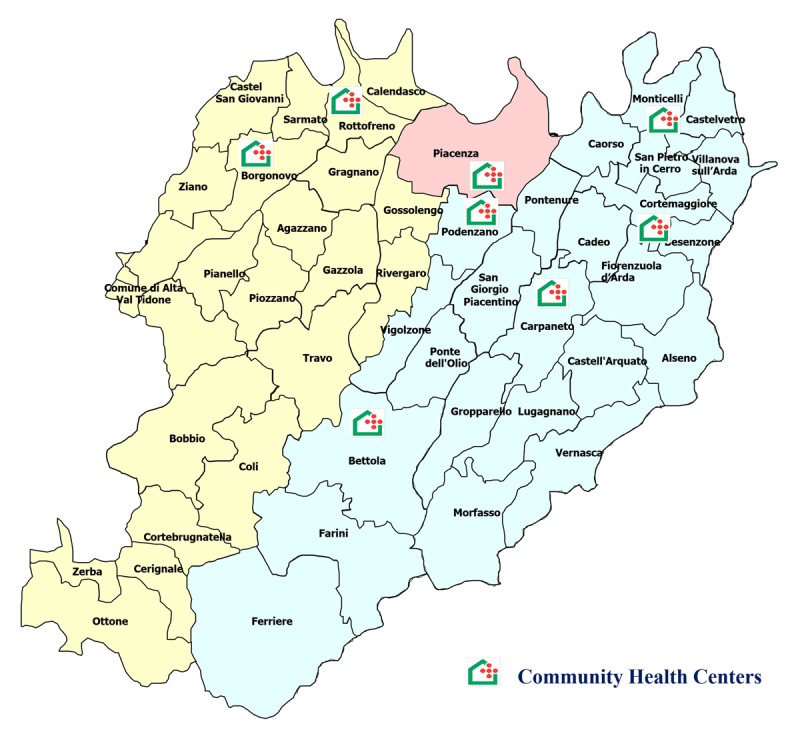
CHCs in the province of Piacenza.

The interviews engaged many variegated participants, comprising six Organizational Managers of all eight Community Health Centers (OMCHC) located in the area of Piacenza (Figure 1), two primary care directors from the LHA, two directors of municipal social services responsible for social and health planning, two representatives from Consultative Committees (CC), and eight representatives from local voluntary organizations. The interviews lasted between 60 and 100 minutes and explored various topics, including the interactions between CHCs and the local welfare ecosystem, encompassing municipalities, schools, and organizations representing patients and caregivers (see Table 1).

**Table 1:** Typology, method, role of interviewees, duration, and role description.


TYPE	METHOD	ROLE	DURATION	ROLE DESCRIPTION

Healthcare Provider	Semi-structured Interviews	Primary Care Director of the Piacenza LHA 1	60 mins	Coordinates health promotion, assesses needs, aids the vulnerable, integrates care, and supports CHC-focused local planning.

		Primary Care Director of the Piacenza LHA 2		

		OMCHC 1	100 mins	Coordinates the CHC’s operational plan, engaging volunteers and citizens, supporting monitoring, and managing stakeholder relations, logistics, and service operations.

		OMCHC 2		

		OMCHC 3		

		OMCHC 4		

		OMCHC 5		

		OMCHC 6		
	
Social Care Provider		Social Services Director 1	90 mins	Coordinates support measures for families, youth, and vulnerable groups, develops services for older adults and those with reduced autonomy, combats social exclusion, and collaborates with volunteers and the LHA in social-health planning.

		Social Services Director 2	60 mins	
	
Voluntary Organizations		CC President 1	60 mins	A citizen representative who monitors, supports, and proposes improvements to healthcare based on user-perceived quality.

		CC President 2		

		Diabetic Association of Piacenza President	60 mins	Citizens and patients offering free support—informational, emotional, logistical, and practical—to others and their caregivers.

	Focus Group	Diabetic Association of Piacenza	120 mins	

		Association for Cerebral Stroke of Piacenza		

		Italian Red Cross of Piacenza		

		Italian Association for Organ Donation of Piacenza		

		Alzheimer Association of Piacenza		

		Italian Multiple Sclerosis Association of Piacenza		

		Association for Active Aging of Piacenza		

		Bone Marrow Donor Association of Piacenza		


In February 2022, a focus group was conducted using Service Design techniques, as outlined by Patricio et al. [44]. The purpose of this focus group was to gather diverse experiences and opinions related to the research topic [45]. Participants were selected based on their expertise and professional distinctions, with the support of primary care directors from the Piacenza LHA and the president of CC.

The decision to use a focus group as a data collection method was driven by fostering participant interaction, encouraging open discussions, and facilitating debate. Along with the deep criticism about the LHA and other health agencies, many proposals to improve collaboration between voluntary organizations and between them and possible partners came to the fore. All interviews and focus group sessions were fully transcribed and analyzed using qualitative analysis software, specifically Atlas.Ti. Our study employed practical thematic analysis [46], which is a widely used and flexible method in health services research. This approach focuses on extracting meaningful insights from the experiences of patients, care partners, and clinicians, without relying on complex theoretical frameworks. While it can support theory-driven analysis, its strength facilitates accelerated, pragmatic investigations grounded in real-world health environments. The interview analysis aimed to identify four key areas of activity emerging from the literature: prevention and health promotion, detecting unmet needs and providing caregiver support, collaborative initiatives, and creating spaces for teamwork.

Throughout the research process, we conducted numerous face-to-face and online interviews with LHA directors and representatives from local voluntary organizations involved in the project. These engagements yielded valuable insights into the operations of the CHCs and the region’s diverse stakeholders, including voluntary organizations and educational institutions. The narrative material from these driven interactions was transcribed and analyzed, with selected excerpts of direct discourse included to substantiate our arguments.

### Implementing Community Health Centers: The Case of Emilia Romagna Region

In 2010, the Emilia-Romagna Regional Council issued Resolution No. 291/2010, outlining regional Community Health Centers (CHCs) guidelines to standardize planning, construction, and management across LHAs, identifying CHCs as a regional health priority [47]. Designed to integrate health and social services, CHCs aim to meet citizens’ needs comprehensively. In 2016, updated directives emphasized horizontal integration and collaboration among healthcare professionals, territorial departments, hospital services, and municipal social services, as well as with citizens, caregivers, voluntary organizations, Consultative Committees, and associations [47,48].

This collaborative, multi-stakeholder approach seeks to address emerging territorial needs through personalized, human-centered care [49,50], now seen as essential to advanced health systems [51]. Including third-sector actors, schools, and local agencies was expected to enrich CHC services. However, reforms have struggled in practice [52], particularly in realizing the envisioned community-based model. CHC activities remain diagnosis- and treatment-focused, underutilizing social, educational, labor, and neighborhood resources [53,54].

A systemic, holistic approach linking institutional services with voluntary and informal family-based support is lacking [55]. Integration is further limited by insufficient social services within CHCs and the absence of dedicated community liaison professionals [56]. Citizen participation, intended as central to CHCs, has faced similar barriers. Though voluntary organizations were meant to co-plan services and assess outcomes [52], CHCs have yet to become spaces for meaningful community involvement. Consultative Committees (CCs) were established to promote citizen input, yet their influence at the planning stage remains limited.

Moreover, weak communication between LHAs, volunteer groups, and technical bodies has hindered the coordination of voluntary services [52]. As a result, CHC social activities are reactive—focused on individual requests—rather than proactive and community-driven. This reinforces a technical, health-centered dynamic, limiting CHCs’ potential to foster social support, participation, and broader well-being.

### The Role of Volunteer Organizations in the CHC

Third-sector Organizations (TSOs) and volunteers are pivotal in designing and delivering citizen-centered, sustainable health and well-being services [57]. Their involvement helps to address resource constraints within healthcare systems. They assume diverse roles: as strategic partners, service providers, community connectors, and liaisons between healthcare and social systems. However, their involvement in intersectoral collaboration often lacks influence, leading to challenges in transitioning between their roles as providers or partners. Achieving authentic and effective collaboration requires a shift in the institutional mindset of professionals towards creating integrated care teams, which can be a complex and demanding process [58].

Despite the challenges associated with implementing the reform, volunteer organizations could play a crucial role in developing the CHC model, as in other primary care services [59,60]. A survey by the Department of Health Policies and Territorial Welfare of Emilia-Romagna revealed that voluntary organizations were active in approximately 70% of the CHCs in the region [61]. Their involvement encompasses many activities, including welcoming and guiding patients, promoting health initiatives, providing accompaniment services, and organizing self-help groups. However, these collaborative efforts have been significantly affected by the COVID-19 pandemic, resulting in the reduction or suspension of many activities. Since then, signs of recovery have emerged.

Data from the Center for Volunteer Services (CVS) indicate a substantial presence of volunteer organizations in the Piacenza area [62]. While these organizations possess considerable expertise, they have significant potential for improvement, particularly regarding their ability to reach individuals who are yet to utilize the services offered by the CHC.

The following section details the primary activities undertaken by volunteers in the Piacenza area, highlighting their independent efforts and collaborative initiatives with CHCs. In the following section, we address the main existing challenges and propose areas for further research.

## Results

Our findings lead to the categorization of voluntary organizations’ primary activities into four key areas: (i) prevention and health promotion, (ii) identification of unmet needs and caregiver support, (iii) collaborative initiatives between voluntary organizations and CHCs, and (iv) the creation of spaces for teamwork. In Piacenza, volunteer organizations and patients actively engage in various prevention and health promotion initiatives, frequently operating without direct support from the LHA or municipal authorities.

The following section will detail volunteers’ primary activities, highlighting their independent efforts and collaborative endeavors with CHCs or within other healthcare premises.

### Prevention and health promotion

One of the crucial activities promoted by voluntary organizations is the coordination of diabetes screening days, which often necessitates financial support from volunteers to engage non-volunteer doctors and specialists. For these programs to achieve optimal effectiveness, seamless integration with the services provided by the LHA is essential, including sharing the data collected from screenings. A primary challenge for these organizations lies in enhancing their capacity to involve new patients, promote treatment adherence, and assist individuals during the initial stages of accepting their diagnosis.

Voluntary organizations also organize awareness-raising meetings that focus on a multitude of health topics. These meetings frequently featured internationally renowned specialists, thereby facilitating the dissemination of advanced knowledge.

“When we organize screening days, we invite diabetes specialists from major hospitals. Having internationally renowned experts speak generates interest.” (Voluntary Organization 1)

Moreover, volunteers are actively involved in primary and secondary schools, emphasizing the importance of health promotion among younger individuals. Schools serve as effective channels for encouraging healthy lifestyles [63,64] and often collaborate with Piacenza’s CHCs and voluntary organizations. Volunteers play an important role in developing preventive initiatives and spreading awareness about various health topics such as proper dietary practices, the significance of blood or bone marrow donation, and early symptom recognition for diseases such as multiple sclerosis.

“When a child has diabetes, we support schools with doctors, train teachers, and engage students from first to fifth grade. Regular visits build familiarity, making communication easier over time.” (Voluntary Organization 1)

“Regrettably, part of our effort to raise awareness among young people is to help them identify their symptoms earlier. The diagnosis of multiple sclerosis typically occurs 3–4 years after the initial symptoms appear; therefore, spreading information is crucial. This is partly because even doctors often take a long time to diagnose.” (Voluntary Organization 6)

Piacenza’s voluntary organizations are frequently present in schools to foster long-term changes in health behaviors. While cooperation with local schools has been highly successful, the COVID-19 pandemic has disrupted numerous ongoing projects, particularly those involving schools in less-populated areas.

### Detecting Unmet Needs and Providing Caregiver Support

Neurodegenerative diseases significantly impact the psychological and social well-being of patients’ families and caregivers, as highlighted by numerous studies [65,66]. However, volunteers currently report limited or no support from institutions for dementia, which can have serious repercussions. In Piacenza, volunteer organizations frequently play a crucial role in identifying and reaching out to vulnerable individuals who lack access to available services.

“Despite rising from 1,000 to 5,000 patients, Piacenza still lacks an Alzheimer’s center. Outdated approaches persist; people remain disoriented. A focused community project and awareness campaign are urgently needed.” (Voluntary Organization 5)

Lack of recognition of informal support may lead to caregivers’ frustration and diminish their willingness to engage with institutional actors. Therefore, there is a pressing need for enhanced collaboration between CHCs and volunteers. Voluntary organizations strongly emphasize the importance of integrating psychological support for the patients involved in their programs.

“The psychologist is key in re-engaging isolated diabetes patients. Expanding support—especially for type 1 diabetics—is vital for truly integrated, team-based care” (Voluntary Organization 1)

Healthcare professionals recognize the necessity of broadening informal networks to address the challenges posed by an aging population, and advocate for increased integration between healthcare and social interventions, particularly the involvement of nurses. Nurses, particularly a new professional role called “community nurse,” play a pivotal role in advancing the objectives of CHCs.

### Collaborative Initiatives Between Voluntary Organizations and CHCs

Even if the reform was not fully implemented and problems arose in interacting with voluntary organizations, many projects and initiatives have been developed through collaborations with some CHCs in the Piacenza area. These projects primarily focus on promoting health and well-being through preventive activities, with goals jointly established by CHC professionals and representatives from third-sector organizations within the CCs. For instance, CCs played a pivotal role in recruiting volunteer organizations for a project designed to develop a service guide for one of the CHCs in the district. In collaboration with the administrative staff, these volunteer organizations created and distributed a guide tailored to meet the community’s specific needs.

“We engaged volunteer organizations to gather their input on how they would prefer the brochure to be structured, what content it should include, and what would make it easier to read and quickly identify the topics most relevant to their interests.” (CC President 1)

However, research findings reveal an uneven distribution of these collaborations across the LHA territory. Interestingly, CHCs in less populated areas, with fewer services, show more significant efforts in promoting projects that involve third-sector actors and engage the local community, compared to others with a higher population density and where a greater variety of services are available.

The CHC’s deep understanding of the local context and its specific needs is crucial for successfully giving birth to volunteer activities in the area and structuring them into organizations. Local CHCs organized community meetings and field training sessions, fostering collaboration and enhancing the impact of these organizations on the community.

“Field training enhanced professional engagement and revealed a wide network of voluntary organizations. Through joint sessions with CHCs, volunteers, GPs, and social services, we co-developed a collaborative guide for community care.” (OMCHC 3)

Interviewees reported that gaining recognition of the CHC model among citizens, general practitioners, and pediatricians often required considerable time and effort. Consequently, this has posed challenges in engaging volunteer participants and in making decisions regarding their involvement in community development initiatives led by the CHC. Moreover, motivating healthcare professionals to engage with local communities actively is particularly challenging, especially given that a significant percentage of the workforce is approaching retirement age. Cultivating increased awareness and willingness among professionals to collaborate with volunteers remains a significant hurdle.

### Creation of spaces for teamwork

The interviews revealed considerable inflexibility in the spatial organization of the CHCs, particularly concerning the physical proximity of professionals, including health and social workers, and voluntary organizations. The absence of dedicated meeting spaces hinders the model’s ability to foster effective teamwork among these groups. Several interviewees suggested the establishment of a social desk within the CHC, which would serve as an integrated access point for health and social services. This would enhance the coordination and alignment of activities and services between health and social professionals. Additionally, providing suitable spaces and equipment for voluntary organizations active in the health and social fields might help support patients and their families by offering practical information and psychological assistance.

One interviewee emphasized the critical role of a volunteer-staffed listening desk, which would provide essential practical and medical information to family members following a patient’s discharge, as highlighted by Petrecca et al. and Driessen et al. [67,68]. The absence of such resources highlights their significance.

“In crises, such as a woman suddenly managing the family business after her husband’s disability, voluntary organizations offer essential guidance. The listening desk addresses this need for support.” (Primary Care Director of the Piacenza LHA 1)

Another interviewee suggested that having a representative from each voluntary organization within the CHC facility would significantly enhance networking and collaboration among these entities through physical proximity:

“Including voluntary organizations in the CHC would streamline communication, allowing for more efficient information sharing and resource allocation among the different entities.” (CC President 1)

## Discussion

This research underscores the critical role of volunteers in bridging the gap between patients and essential support services, as highlighted by Gaber et al. [12]. Health and social services often encounter situations encompassing healthcare and broader social needs, including housing support, economic assistance, and guidance for caregivers. While some of these needs are recognized and reported to social services provided by the municipality, others remain unaddressed because they are unexpressed or lack solutions within the local welfare system. Volunteer organizations serve as “important agents of social change by detecting unmet needs” [69] and as intermediaries connecting citizens to institutions. Particularly in isolated areas, local volunteers, who are well-known within their communities, play a vital role in identifying and reporting cases of social deprivation to social service providers.

Embracing a preventive medicine approach incorporating social welfare interventions is gaining increasing recognition [70]. This approach is instrumental in mitigating the adverse effects of complex issues such as loneliness and chronic illness, which tend to become progressively more challenging to manage over time [71]. From a healthcare perspective, dementia is often unrecognized by family members, leading to delayed diagnosis. Loneliness among patients and their families dealing with dementia emerged as a prevalent concern during interviews. Stakeholders acknowledged this challenge within the aging population, emphasizing the necessity for comprehensive interventions and advocating their implementation.

### Coordination among voluntary organizations

The lack of formal coordination among voluntary organizations operating in the social and health sectors is a common issue in the Piacenza area. The results revealed several obstacles to coordination among third-sector entities, such as organizational individualism, competition for limited resources and funding (primarily from the local foundation), low motivation among voluntary organizations to address complex issues, and the absence of dedicated physical workspaces. Despite these challenges, the interviews revealed that small groups of voluntary organizations sometimes collaborate on specific projects and activities within precise time and space boundaries. There is a growing demand for a coordination board, potentially led by various stakeholders such as the municipal administration, Social Services, the Volunteer Service Center (a regional body supporting local voluntary organizations), and the Piacenza CC. Such a board could enhance communication during service planning and unite volunteers interested in specific thematic areas, aligning coordination efforts with people’s interests.

### Economic Acknowledgment

Currently, neither the municipality nor the LHA provides financial support for volunteer projects, despite these activities often complementing the services offered by social and health systems. Many organizations rely on annual funding from local foundations, which have long played the role of social innovators, addressing the deficiencies of public agencies and acting as reliable substitutes across various welfare domains [72].

The current system emphasizes competition among organizations rather than promoting collaboration in accessing funding opportunities. Consequently, similar and complementary activities are often pursued in isolation. Some voluntary organizations, especially small ones, require support to access public funds because of cumbersome bureaucratic application procedures or a lack of knowledge about the application process.

### Participation in health service monitoring

The three CCs collaborate closely with Primary Care Directors in the Piacenza area to oversee and improve healthcare services. These committees actively participate in various initiatives, assessing the health authorities’ efforts to enhance communication and relationships with citizens across Piacenza LHA districts.

Interview feedback indicates that CCs effectively monitor service quality and address specific issues, such as improving patient care, food quality, and mobility inwards, like orthopedics and medicine. They have also advocated for better accessibility and service delivery in the CHC, including integrating volunteers to enhance patient support, promoting improved management of chronic diseases, such as diabetes care, and partnering with local social and health stakeholders. However, some challenges remain to be addressed. First, there is a notable disparity in activity levels among districts. Some committees are highly active and hold regular meetings, while others have slowed down because of leadership changes. Second, there is a need for greater representation of voluntary organizations within CCs to improve their effectiveness. Raising awareness among users about CCs’ roles and contributions in monitoring healthcare service quality is crucial because it fosters transparency and trust between the community and healthcare providers. When users understand CCs’ advocacy efforts, such as improving service delivery or addressing patient concerns, they are more likely to participate in the feedback process actively. Finally, some interviewees expressed uncertainty regarding the capacity of CCs to support broader development and implementation of the CHC model.

### Enhancing the social role of community nurses as first contact with voluntary organizations

This research highlights the evolving role of nurses and the increasing expectations placed on them within the CHC model, as noted in previous studies [73]. Nurses are expected to demonstrate strong interpersonal skills to address patients’ health needs and the broader social and supportive aspects of care. Concepts like “trust” and “empathy” are frequently emphasized when considering nurses’ current and future roles, particularly with the introduction of a new professional role, the “community nurse.” These community nurses were designed to work within and for local communities, engaging with individuals who might otherwise lack access to care [74]. Their role is instrumental in CHCs, as they can independently address community-specific needs and foster new collaborations with voluntary organizations, strengthening the overall support network.

This organization is comparable to the Social Prescribing (SP) scheme introduced by the UK National Health Service in 2019 [75,76]. However, there were two key differences. In the SP scheme, the role of link workers is typically filled by social workers rather than by nurses. More importantly, the involvement of third-sector organizations and volunteers in SP is integral, forming part of a “social prescription.” In contrast, volunteer participation is considered optional in CHCs. The UK Social Prescribing Model is currently being piloted in several Italian cities, such as Trento, Verona, and Bologna. In theory, CHCs are well suited to host SP services, providing patients access to a wide range of activities offered by voluntary and community sectors. However, the evolution of the community nurse role or the potential introduction of roles akin to link workers is still under discussion.

Volunteer organizations embody a shared commitment to community welfare, bridging healthcare and the broader community through active engagement, robust local networks, and addressing diverse needs [77]. Within the CHC model, these organizations are integral to a comprehensive framework that brings together institutions, professionals, and services to deliver coordinated, community-centered care, emphasizing prevention and primary health interventions. Community well-being encompasses physical, mental, and social dimensions shaped by factors, such as healthcare accessibility, service effectiveness, active community participation, and individual empowerment. These elements interconnect and dynamically influence each other, creating the complex tapestry of community-based healthcare (Table 2).

**Table 2:** Interactions and dynamics of community-based healthcare.


FROM	Volunteer Organizations	Community Health Centers	Community

To			

**Volunteer Organizations**		Enrich the CHC with insights, resources, and community perspectives.	Empower individuals and foster healthy behaviors and culture

**Community Health Centers**	Gain expertise, access community networks, and address health needs.		Deliver accessible services, enhance well-being, and reduce disparities.

**Community**	Reinforce their importance, motivate engagement, and support health and social improvements.	Drive demand, align services, and adapt strategies for improved health.	


## Conclusions

Voluntary organizations in Piacenza are crucial for providing healthcare and social care support. However, significant challenges remain regarding their integration within the local welfare ecosystem, particularly in the “new” CHCs [55]. Addressing these challenges is essential for successfully implementing the CHC model, which emphasizes the active involvement of voluntary organizations and the local community in enacting practical changes, as noted by Ingrosso [56].

In the context analyzed, bridging the gap between formal and informal care requires establishing local partnerships among social and healthcare professionals, citizens, service users, patients, and policymakers. These partnerships aim to mobilize individual and collective efforts to address various challenges [77]. However, their effectiveness often remains ideological and requires practical improvement to achieve tangible results.

The exploration of the complex interactions and dynamics inherent in the community-based healthcare system of Piacenza showed that while some critical dimensions of this field have been illuminated, others warrant deeper investigation for future research that includes volunteer activities:

- **Generating empowerment amplification** [78,79]: Investigating the mechanisms by which volunteer organizations empower communities and understand the factors that enhance or hinder their capacity to mobilize resources and catalyze health-related behaviors. Additionally, exploring innovative methods for measuring the lasting impact of community empowerment on long-term health outcomes could yield valuable insights.
- **Creating optimal integration strategies** [12]: Fostering seamless integration between volunteer organizations and the broader CHC ecosystem. Our research underscores that successful integration relies on efficient decision-making, resource sharing, and coordination models. These strategies can enhance the delivery of holistic healthcare services by aligning efforts and maximizing the impact on both formal healthcare providers and volunteer groups.
- **Ensuring sustainability and scalability** [80]: Examining the sustainability and scalability of resources produced by volunteer organizations. Future research should assess strategies to maintain the vitality of these organizations over time (recruiting new volunteers that can replace the existing ones, quite aged), ensuring continued community engagement and extending their influence beyond local contexts.
- **Adapting to uncertainty** [81]: Given the ever-evolving nature of the healthcare landscape, future research could focus on strategies that enable volunteer organizations and the healthcare system to dynamically adapt to unforeseen changes. This could involve investigating innovative approaches to community engagement, health communication, and flexible service delivery.

It is essential to acknowledge the limitations of this study. First, the study was conducted during the COVID-19 pandemic, significantly restricting its scope owing to limitations in accessing CHCs. While most interviews were conducted online [82], focus group sessions were conducted in person. Second, a substantial challenge emerged from the transitional phase of the CHC regulations. During the final stages of our research, a new primary care reform was initiated as part of the National Recovery and Resilience Plan, resulting in the development of community health centers known as ‘Case della Comunità.’ This new model involves less engagement from voluntary organizations than the previous ‘Case della Salute.’ Third, within this complex environment, our study was limited to a relatively small number of voluntary organizations. This constraint reduced the diversity of perspectives and experiences that we could capture, potentially limiting the generalizability of our findings.

Furthermore, the organizations involved had limited operational specialization, which may not fully represent the broader sector’s range of capabilities and expertise. Lastly, their spatial distribution was concentrated in specific areas, potentially skewing the results by omitting region-specific challenges or successes [83]. However, given the limited research on the role of volunteer organizations in implementing new community care services within the Italian context, our study offers a modest but valuable contribution to this ongoing debate.
