# Correction: Exploring the Role of Volunteer Organizations in Developing Italy’s Community-Based Care Model

**DOI:** 10.5334/ijic.10219

**Published:** 2025-12-01

**Authors:** Federico De Luca, Giuliana Costa, Cristina Masella

**Affiliations:** 1 Department of Management Engineering, Department of Design, Politecnico di Milano, Milan, Italy; 2 Department of Architecture and Urban Studies, Politecnico di Milano, Milan, Italy; 3 Department of Management Engineering, Politecnico di Milano, Milan, Italy

**Keywords:** community-based care, volunteer organizations, primary care, community health center

## Abstract

This article details a correction to: De Luca F, Costa G, Masella C. Exploring the Role of Volunteer Organizations in Developing Italy’s Community-Based Care Model. *International Journal of Integrated Care*. 2025;25(3):3. DOI: https://doi.org/10.5334/ijic.7881.

## Correction

The article “Exploring the Role of Volunteer Organizations in Developing Italy’s Community-Based Care Model” [1] was originally published with a reference list containing several erroneous references. These errors were introduced by the misapplication of a style-conversion tool in conjunction with referencing software. The corrected references have been subsequently provided by the corresponding author and replaced in the published version.
